# NF-κB-Like Signaling Pathway REL2 in Immune Defenses of the Malaria Vector *Anopheles gambiae*

**DOI:** 10.3389/fcimb.2017.00258

**Published:** 2017-06-21

**Authors:** Suzana Zakovic, Elena A. Levashina

**Affiliations:** Vector Biology, Max-Planck Institute for Infection BiologyBerlin, Germany

**Keywords:** *Anopheles gambiae*, *Plasmodium*, NF-κB signaling, IMD, REL2 pathway, malaria, vector biology, innate immunity

## Abstract

The blood feeding requirements of insects are often exploited by pathogens for their transmission. This is also the case of the protozoan parasites of genus *Plasmodium*, the causative agents of malaria. Every year malaria claims the lives of a half million people, making its vector, the *Anopheles* mosquito, the deadliest animal in the world. However, mosquitoes mount powerful immune responses that efficiently limit parasite proliferation. Among the immune signaling pathways identified in the main malaria vector *Anopheles gambiae*, the NF-κB-like signaling cascades REL2 and REL1 are essential for eliciting proper immune reactions, but only REL2 has been implicated in the responses against the human malaria parasite *Plasmodium falciparum*. Instead, constitutive activation of REL1 causes massive killing of rodent malaria parasites. In this review, we summarize our present knowledge on the REL2 pathway in *Anopheles* mosquitoes and its role in mosquito immune responses to diverse pathogens, with a focus on *Plasmodium*. Mosquito-parasite interactions are crucial for malaria transmission and, therefore, represent a potential target for malaria control strategies.

## Introduction

Mosquitoes are vectors of human infectious diseases with immense importance for public health. Malaria, caused by the *Plasmodium* protozoa, is the deadliest disease transmitted by *Anopheles* mosquitoes. *Plasmodium* development in the mosquito takes about 3 weeks. A series of mosquito factors affect malaria transmission, among them female longevity, nutritional fitness and efficient immune responses. The ookinete is by far the most fragile parasite stage, attracting the majority of immune responses. It swiftly develops from the sexually created zygote, and its task is to escape the dangerous gut environment by traversing the unicellular epithelium and to hide beneath the basal lamina that lines the mosquito midgut. If successful, ookinetes transform within the second day into vegetative protective oocysts that in a fortnight give rise to thousands of sporozoites that migrate and invade the salivary glands to be ready for a new transmission. Vector-parasite molecular interactions have been studied mostly in the laboratory model of infections of *A. gambiae*, the major malaria vector in the sub-Saharan Africa, with the rodent malaria parasite *P. berghei*. Although rodent and human parasites have similar invasion strategies in the insect vector, their elimination is mediated by two distinct nuclear factor-κB (NF-κB) immune pathways, REL2/Imd and REL1/Toll. NF-κB, initially discovered for its DNA-binding activity to an immunoglobulin-κ light chain enhancer in B lymphocytes, emerged as the central regulator of immune responses in animal kingdom (Sen and Baltimore, 1986). In *Anopheles*, experimental activation of REL2 aborts development of *P. falciparum* ookinetes, while constitutive induction of REL1 kills rodent parasites (Frolet et al., 2006; Garver et al., 2009). However, in both cases the molecular mechanisms of parasite recognition and killing remain unknown. In this review, we discuss immune responses of *A. gambiae*, with a focus on the REL2 signaling pathway, which is believed to be the major regulator of mosquito immune responses against human malaria parasites.

## Immune signaling and pathogen recognition

The Toll and Imd pathways in *Drosophila* regulate expression of hundreds of infection-inducible genes. While the Toll pathway, initially described in *Drosophila* embryonic development, is essential for defenses against Gram-positive bacteria and fungi (Lemaitre et al., 1996; Rutschmann et al., 2002), the Imd pathway orchestrates responses against Gram-negative bacteria and viruses (Kaneko et al., 2004; Costa et al., 2009).

**Figure 1:**
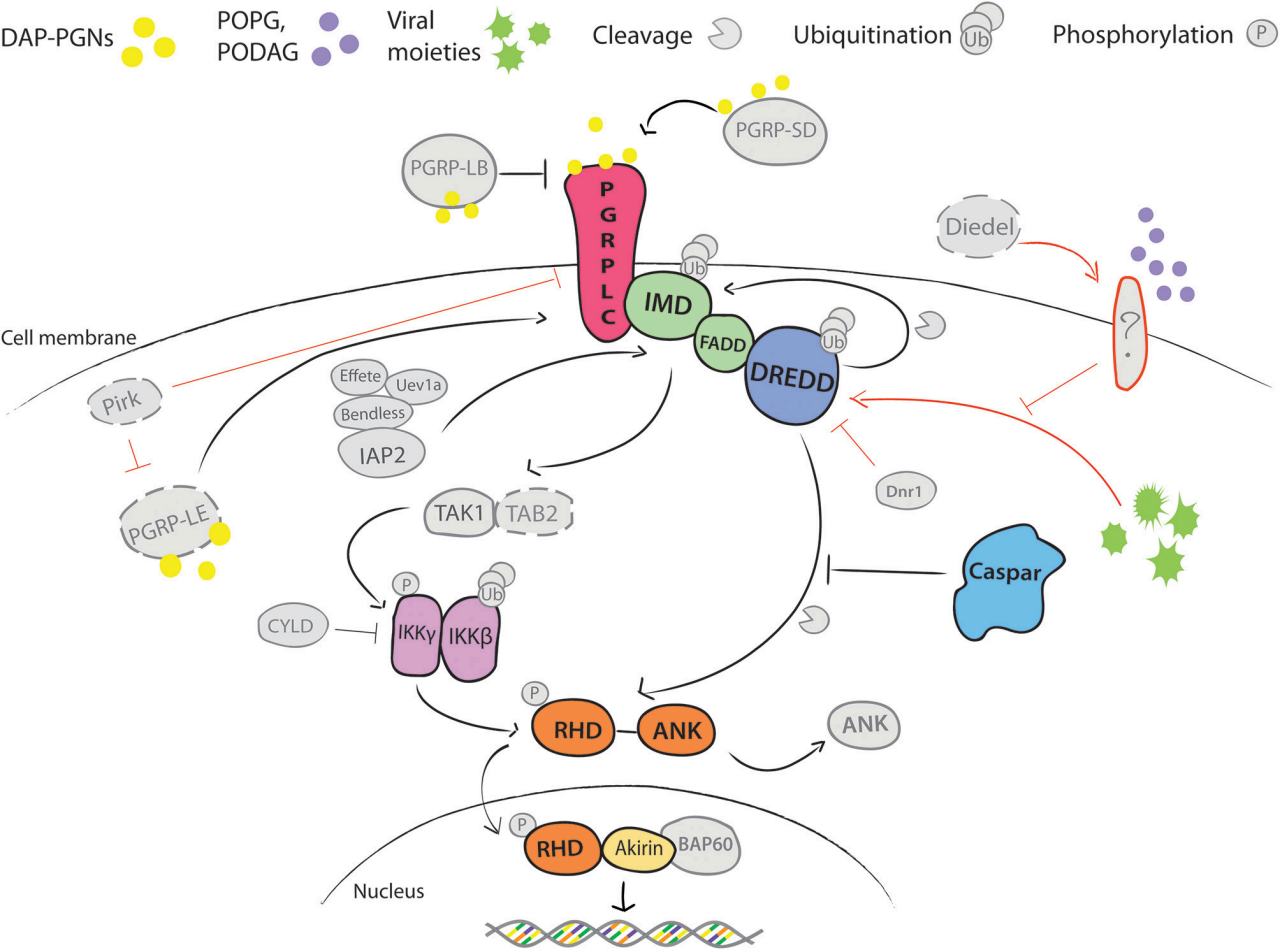
Schematic overview of Imd pathway in *Drosophila melanogaster*. DAP-type PGNs trigger the activation of Imd signaling by direct interaction with the immune cell. Of Peptido-Glycan Recognition Proteins (PGRPs) that bind these pathogen-derived molecules, transmembrane PGRP-LC is the main receptor linked to activation of Imd pathway (Choe et al., 2002; Gottar et al., 2002). Its activity is enhanced in circulation by secreted PGRP-SD and intracellularly by the cytosolic PGRP-LE (Takehana et al., 2002; Iatsenko et al., 2016). Both proteins can directly bind DAP-PGNs and promote PGRP-LC activity. Another extracellular PGN-binding protein PGRP-LB antagonizes PGRP-LC activity by scavenging PGNs in circulation (Zaidman-Rémy et al., 2006). PGN binding induces conformational changes in PGRP-LC that promotes recruitment of the death domain-containing proteins Imd, FADD and DREDD caspase from the nucleus to the plasma membrane, and it is followed by subsequent polyubiquitination of DREDD by ubiquitin E3 ligase IAP2, cleavage of Imd by DREDD and exposure of K63 site for polyubiquitination by IAP2 and E2 conjugating enzymes Bendless, Effete and Uev1a (Paquette et al., 2010; Meinander et al., 2012). The K63-polyubiquitin chains, most likely, serve as activators of TAK1 kinase via the ubiquitin-binding domain of its regulatory protein TAB2 (Paquette et al., 2010). TAK1/TAB2 complex phosphorylates IKK complex, which consists of β and γ subunits. IKKβ further phosphorylates the NF-κB-like transcription factor Relish, while a regulatory IKKγ subunit regulates DREDD-mediated cleavage of Relish (Ertürk-Hasdemir et al., 2009). Relish consists of the Rel Homology Domain (RHD) and the inhibitory ankyrin-repeat rich domain (ANK) (Dushay et al., 1996). DREDD caspase cleaves the ANK domain from RHD. RHD translocates to the nucleus and initiates transcription of target genes. Caspar acts as a negative regulator of the pathway by inhibiting DREDD-dependent cleavage of Relish (Kim et al., 2006). Immunomodulatory cytokine Diedel restrains deleterious non-canonical activation of Imd in presence and absence of viral infection (Lamiable et al., 2016). Receptor that activates the pathway to viruses is not yet known However, epistasis analyses placed Diedel function between Imd and IKKγ, as mutants for both Diedel and Imd were more prone to spontaneous pathogenesis than Diedel/IKKγ double mutants (Lamiable et al., 2016). Additionally, activation of the pathway is held in check by other factors, including CYLD, Dnr1, and Pirk. Finally, transcriptional activity of Relish is regulated at the chromatin level through interactions with a nuclear co-factor Akirin and BAP60 component of Brahma chromatin remodeling complex. Akirin recruits BAP60 complex to promoters of a subset of Relish effector genes and hence regulates their transcription (Goto et al., 2008; Bonnay et al., 2014). Positive and inhibitory interactions are depicted with → and |–, respectfully; black—well established, and red—yet unknown interactions. Color coding highlights our current knowledge on the pathway in *Anopheles gambiae*. Confirmed pathway components are indicated in color. Components depicted in gray represent orthologs identified by genomic searches, but whose function was not experimentally validated. Components in gray with dashed lines are absent in *A. gambiae*.

Immune activation is mediated by recognition of pathogen derived molecules, such as metabolites, nucleic acids, or cell wall components that are released during pathogen growth and division (Vance et al., 2009). Among microbial and host factors that induce Imd signaling, the best characterized is the DAP-type peptidoglycan (DAP-PGN), a cell wall component of Gram-negative bacteria. DAP-PGN recognition at the cell surface is mediated by transmembrane peptidoglycan recognition proteins (PGRPs) (Choe et al., 2002; Gottar et al., 2002; Figure 1). In contrast, activation of the Toll pathway is initiated by binding of circulating recognition complexes to the lysine-type PGNs or glucans (Gobert et al., 2003; Leulier et al., 2003). This binding triggers downstream serine protease cascades, leading to processing and binding of the endogenous factor Spaetzle to the transmembrane receptor Toll (Morisato and Anderson, 1994; Schneider et al., 1994). Activation of both pathways culminates in the phosphorylation and release of the NF-κB-like transcription factors Dif, Dorsal and Relish from the inhibitors, and their translocation into the nucleus. The release of the Imd transcription factor Relish involves Caspar, which in its inactive state prevents cleavage of the Relish inhibitory domain (Kim et al., 2006; Figure 1). Phosphorylation of Cactus, the negative regulator of the Toll pathway, leads to its degradation and release of the transactivators Dif and Dorsal (Wu and Anderson, 1998).

The Imd role in *Drosophila* immunity has been recently extended to antiviral responses. Surprisingly, instead of protection, constitutive activation of the Imd pathway by depletion of a secreted cytokine-like molecule Diedel, enhances viral pathogenesis (Lamiable et al., 2016). Although the underlying mechanism is not entirely clear, in the absence of Diedel, viral infection triggers the pathway through an alternative, non-canonical cytoplasmic route by bypassing the function of the PGRP receptors (Lamiable et al., 2016). Similarly, non-canonical activation of this pathway was also reported in ticks that lack transmembrane PGRP-LC and death domain proteins, Imd and FADD, but feature a conserved ubiquitination module, Relish and Caspar (Figure 1). The tick pathway is induced by lipid components of membranes specific to PGN-deficient bacteria (Shaw et al., 2017). Importantly, the same lipids also induce the Imd pathway in Drosophila cell lines, suggesting that non-canonical activation of the Imd pathway may be evolutionarily conserved (Shaw et al., 2017).

## REL2 pathway in *A. gambiae*

The vast knowledge that accumulated on the immune signaling in Drosophila has served as a blueprint for studying *Anopheles* mosquitoes. Sequencing of the *A. gambiae* genome benefited identification of the conserved components of the pathway (Christophides et al., 2002; Holt et al., 2002). However, in spite of considerable interest and potential importance in antiparasitic responses, surprisingly little is known about the targets of the Imd/REL2 pathway in *Anopheles*.

Genomic searches in *A. gambiae* identified three potential receptors: PGRP-SD, PGRP-LB and PGRP-LC. PGRP-SD has not been characterized, whereas functional analysis of PGRP-LB did not reveal its role in mosquito survival upon bacterial infections (Meister et al., 2009). Instead, the structure and function of PGRP-LC were characterized in a great detail (Meister et al., 2009). PGRP-LC encodes three splice variants (LC1-3) that differ in the organization of their extracellular PGN-binding domains. Structural modeling uncovered the potential of all three isoforms to bind both types of PGNs, highlighting a striking difference between the mosquito PGRP-LC with broad sensing capacities and the *Drosophila* PGRP-LC, which binds exclusively DAP-PGNs. The broad specificity of PGN binding of the *Anopheles* PGRP-LC was further substantiated by functional analyses that demonstrated equally critical role of the receptor in mosquito survival to Gram-negative and Gram-positive bacteria (Meister et al., 2009). Although infections with both bacteria induced expression of genes encoding antimicrobial peptides (AMPs) Cecropin1 and Defensin1, their transcriptional induction was PGRP-LC independent (Meister et al., 2009), therefore, the mechanisms underlying the PGRP-LC-mediated resistance to bacteria remain to be elucidated.

PGRP-LC plays an important role in regulating proliferation of the mosquito microbiota after blood feeding (Meister et al., 2009) and may, in big part, explain the role of the pathway in modulating development of *Plasmodium* parasites. Indeed, similar to the phenotype of *PGRP-LC* silencing, clearing mosquito microbiota by antibiotics prior to infections increases *Plasmodium* loads, whereas feeding mosquitoes with bacteria boosts their resistance to *Plasmodium* in a *PGRP-LC*-dependent manner (Meister et al., 2009). Therefore, it is possible that the REL2 pathway is activated after blood feeding by massive bacterial proliferation, whereas *Plasmodium* parasites are simple bystanders in this process and do not directly induce mosquito immune responses. Further transcriptomics studies examined mosquito responses to infections with *P. falciparum* and *P. berghei* and identified species-specific patterns of gene expression (Dimopoulos et al., 2002; Dong et al., 2006). Nevertheless, a significant overlap observed in the responses to bacterial and *Plasmodium* infections provides further support to the hypothesis that REL2 modulation of *Plasmodium* development may be triggered by the PGRP-LC-mediated recognition of bacteria.

Regardless of the trigger, it is expected from the *Drosophila* model that conformational changes of PGRP-LC will recruit the death-domain containing receptor-adaptor complex (Imd, FADD and DREDD), which will, via the TAK1/TAB2 complex, activate the IKK signalosome and inhibit the negative regulator Caspar (Figure 1). Relish activation is achieved by phosphorylation by the IKK signalosome and by cleavage of the inhibitory ankirin domain by the DREDD caspase (Figure 1). Transcriptional activity of Relish at the promoters of some genes is further regulated by its nuclear co-factor Akirin. Using RNAi silencing and its effects on *P. falciparum* infections, most of the pathway components were functionally confirmed in *A. gambiae*, except for TAK1, whose depletion did not impact *Plasmodium* development (Meister et al., 2005; Garver et al., 2012; Ramphul et al., 2015).

Further studies of the REL2 pathway identified some mosquito-specific particularities. In contrast to *Drosophila, REL2* in mosquitoes encodes *three* alternatively-spliced isoforms that were first identified in another mosquito species, *Aedes aegypti* (Shin et al., 2002; Antonova et al., 2009). In *A. gambiae*, Meister et al. (2005) described two *REL2* forms: a long transcript (*REL2-F*) coding for the full-length protein consisting of Rel-homology domain (RHD) and ankyrin-rich repeat (ANK), and a short form (*REL2-S*) encoding only RHD. Although authors proposed that the isoforms regulate expression of distinct sets of genes, both isoforms regulate *Plasmodium* development (Garver et al., 2012). Currently, the molecular mechanisms underlying activation of REL2 in *Anopheles* remain unresolved. It is unknown whether REL2 requires proteolytic activation by CaspL1 (*Anopheles* ortholog of DREDD) and how Caspar inhibits its activation. Surprisingly, functional analyses by gene silencing suggested a genetic interaction between Caspar and REL2-S, whereas regulation of REL2-F was not investigated (Garver et al., 2012). These results are inconsistent with the proposed mechanism of Relish inhibition by Caspar in *Drosophila*, where Caspar binding to the inhibitory ankirin domain prevents its cleavage by DREDD. However, as REL2-S lacks the ANK domain, the inhibitory mechanism of Caspar in *Anopheles* remains unclear. Co-silencing of *Caspar* with *Imd, FADD, CaspL1* or *IKK2*, rescued the negative effect of the single *Caspar* knockdown on parasite development, confirming the role of these components in activation of REL2. Of particular interest is the role of Akirin, the nuclear co-factor of REL2, which regulates chromatin conformation and provides access to the promoter regions of a set of effector genes in *Drosophila* (Goto et al., 2008; Bonnay et al., 2014). As Akirin contributes to antiparasitic responses (Da Costa et al., 2014), better understanding of its function and of its target genes should shed light on regulation of *Plasmodium* killing.

So far, abortion of *Plasmodium* development was observed only upon experimentally-induced activation of the NF-κB pathways. Therefore, species-specific elimination of *Plasmodium* parasites by REL2 and REL1 seems to be independent of parasite recognition and may be explained by the activation of distinct sets of effectors. It is also plausible, that *Plasmodium* species differ in their susceptibility to the immune defenses triggered by these pathways. Therefore, understanding the molecular mechanisms underlying the pathway-specific parasite elimination may provide interesting insights into biology of *Plasmodium* species.

## Effectors

Antimicrobial peptides are powerful effectors of innate immunity (Jenssen et al., 2006). They bind and directly kill a broad spectrum of pathogens by disrupting cell membrane integrity (Yeaman and Yount, 2003). Insect AMPs are synthesized by the fat body with some contribution of hemocytes, and are secreted into the hemolymph shortly after infection. In addition, some AMPs are also produced by epithelial cells in a tissue-specific manner (Tzou et al., 2000). Expression of the AMP genes *Drosomycin* and *Diptericins* in the fat body is regulated by Toll and Imd, respectively, whereas both pathways contribute to expression of other AMP genes (e.g. *Defensin, Drosocin, Metchnikowin, Attacins*, and *Cecropins*) (Ferrandon et al., 2007).

Several AMP genes have been identified in *A. gambiae*: *Defensins* (*Def1-5*), *Cecropins* (*Cec1-4*), *Gambicin* (*Gamb*), and *Attacin* (Holt et al., 2002; Mongin et al., 2004). Antimicrobial and antifungal activities of the recombinant Cec1, Gamb, and Def1 peptides were demonstrated *in vitro* against filamentous fungi (Cec1, Gamb, Def1), Gram-negative (Cec1, Gamb) and Gram-positive bacteria (Def,1 Cec1, Gamb) (Vizioli et al., 2000, 2001a,b). *In vivo* silencing of *Def1* increases mosquito susceptibility to Gram-positive bacteria but does not affect development of *P. berghei* (Blandin et al., 2002). At the transcriptional level, however, expression of *Def1* was upregulated by infections with human and rodent parasites (Tahar et al., 2002). Gambicin, the only mosquito-specific AMP, exhibited some activity against *P. berghei* ookinetes *in vitro*, whereas depletion of *Gamb in vivo* increased mosquito susceptibility to Gram-positive bacteria, *P. berghei* and, to a lower extent, to *P. falciparum* (Vizioli et al., 2001a; Dong et al., 2006). Interestingly, transgenic over-expression of Cec1 fused to the Shiva toxin inhibited development of *P. berghei* oocysts in *A. gambiae* (Kim et al., 2004). In spite of these results, the exact role of antimicrobial peptides in the mosquito defenses against *Plasmodium* is still incompletely understood.

Identification of the effectors of REL2 and REL1 is crucial for understanding the specificity of malaria killing in the mosquito. However, only a handful of immune genes in *Anopheles* have been assigned to either pathway. Frolet et al. (2006) did not observe any changes in the expression of AMP genes upon constitutive activation of REL1, leaving an open possibility of their regulation by REL2. *In vitro* studies provided some support of *Cec1* and *Gamb* regulation by REL2 (Meister et al., 2005), but a more recent study *in vivo* suggested a dual regulation of AMP genes by both pathways (Garver et al., 2009).

The complement-like system emerged as a powerful arm of the mosquito immune responses to a broad spectrum of pathogens. The central component of this system, the thioester-containing protein 1 (TEP1), is a major determinant of malaria killing (Blandin et al., 2004; Garver et al., 2009; Molina-Cruz et al., 2012; Nsango et al., 2012). TEP1 binds to the surface of invading *Plasmodium* ookinetes and bacteria, and promotes their killing by lysis and phagocytosis, respectively (Blandin et al., 2004). TEP1 is a highly reactive protein and requires a complex of two leucine-rich repeat proteins [leucine-rich repeat immune protein 1 (LRIM1) and *Anopheles Plasmodium*-responsive leucine-rich repeat 1C (APL1C)] to prevent its precocious activation and precipitation (Frolet et al., 2006; Fraiture et al., 2009). *TEP1* or *LRIM1* co-silencing with *Cactus* completely reverts the refractory phenotype of *Cactus* knockdown in *A. gambiae* infections with *P. berghei* (Frolet et al., 2006). Silencing of *TEP1* also results in higher intensities of *P. falciparum* infections (Garver et al., 2009), however, levels of TEP1 protection against *P. falciparum* vary with the genotype and genetic complexity of *Plasmodium* infections (Molina-Cruz et al., 2012; Nsango et al., 2012). Similar to AMPs, expression of the complement-like genes is regulated by both pathways (Frolet et al., 2006).

Another interesting multi-member protein family with potential roles in mosquito immune responses is the family of fibrinogen related proteins (FBNs or FREPs). It comprises 59 members in *A. gambiae*, 37 in *A. aegypti* and 14 in *D. melanogaster* (Christophides et al., 2002; Dong and Dimopoulos, 2009). Only few FBNs have been functionally characterized. Silencing of *FBN9, FBN22* and *FBN39* impairs mosquito survival upon bacterial infections; depletion of *FBN8, FBN9, FBN30*, and *FBN39* increases mosquito susceptibility to *Plasmodium* parasites, while silencing of *FBN1* decreases parasite loads (Dong and Dimopoulos, 2009; Li et al., 2013; Simões et al., 2017). Little is known about regulation of *FBN* expression, except for *FBN9*, whose expression is regulated by REL2 (Garver et al., 2009). FBN9 binds to malaria parasites and bacteria, thereby exposing them for killing by an as yet unknown mechanism (Dong and Dimopoulos, 2009). Surprisingly, transgenic expression of *FBN9* in the fat body driven by a blood feeding-inducible promoter did not enhance mosquito resistance to *P. falciparum* (Simões et al., 2017). This unexpected result highlights the importance of tissue-specific REL2 regulation, which has not been addressed yet.

## Cellular immune responses

Several independent reports implicated REL2 pathway in the immune responses of hemocytes, the mosquito blood cells. Transcripts encoding the pathway components were identified in the hemocyte-enriched transcriptome and their expression levels were further upregulated by blood meal and by *P. berghei* ookinetes (Baton et al., 2009). Furthermore, hemocytes synthesize proteins whose expression is regulated by the REL2 pathway (e.g., AMPs, TEP1, LRIM1, and FBN9) (Levashina et al., 2001; Baton et al., 2009). Some of these proteins (TEP1, TEP3, PGRP-LC, and LRIM1) also contribute to the efficient phagocytosis of Gram-positive and Gram-negative bacteria (Moita et al., 2005). In *Drosophila*, hemocytes also serve as messengers in inter-organ communication. For example, an amplitude of systemic immune responses induced by localized infections is diminished in hemocyte-depleted fruit fly mutants, revealing hemocyte contribution to the amplification of the fat-body mediated immune responses (Charroux and Royet, 2009; Wu et al., 2012). The reactive oxygen species that act as local triggers of hemocyte activation, also efficiently activate the Imd pathway (Foley and O'Farrell, 2003; Wu et al., 2012), further supporting the potential role of this pathway in hemocyte activation.

## Melanization

The *Anopheles* REL2 pathway negatively regulates melanization, a process of melanin deposition in defense mechanisms (such as wound healing or pathogen killing), metamorphosis and tanning during development. Silencing of *PGRP-LC* and of both *REL2* isoforms not only renders mosquitoes more susceptible to *Plasmodium* but also induces melanization of *P. berghei* ookinetes (Meister et al., 2005, 2009; Frolet et al., 2006). The reverse is observed in *Drosophila*, where the intracellular receptor PGRP-LE is absolutely required for melanization (Takehana et al., 2004). REL1 pathway, on the other hand, promotes melanization in both insect species (Ligoxygakis et al., 2002; Frolet et al., 2006). Surprisingly, simultaneous activation of the REL1 pathway by *Cactus* knockdown and inhibition of the REL2 pathway by REL2 silencing abolishes *Plasmodium* melanization, revealing the complexity in the regulation of this immune reaction and a potential cross-talk between the two pathways (Frolet et al., 2006).

## Conclusions

Although a significant progress has been achieved in identifying the components of the REL2 pathway in mosquitoes, many questions remain unanswered. Little is known about the role of post-translational modifications, such as ubiquitination and phosphorylation, which act as important pathway regulators in *Drosophila*. Moreover, completely unexplored areas are the contributions of the epigenetic modifications acting at the promoter level to fine tune immune responses. Understanding how chromatin conformations modulate expression patterns of effector genes may offer new insights into the complexity and specificity of REL2-mediated immune responses to a broad range of pathogens. However, even before considering the complexity of epigenetic modifications, it is pivotal to characterize the REL2-specific effectors, which are currently only vaguely known; and to address the questions of *Plasmodium* recognition and pathway activation upon infection in order to properly understand the parasite-host interaction and *Plasmodium* killing in the mosquito.

Recent studies in ticks and *Drosophila* discovered a non-canonical cytoplasmic route of pathway activation that bypasses the PGRP receptor-adaptor complex. These observations open new research avenues regarding receptor(s) and molecular mechanisms of pathway activation. The conservation of this non-canonical route in the evolutionarily distant organisms, such as ticks and *Drosophila*, may suggest that PGN recognition by PGRPs and further signal transduction by the death-domain module, appeared later in evolution, after separation of arachnids from insects. Instead, the core pathway from the ubiquitination module via Caspar to Relish seems to be broadly conserved across arthropods. Better understanding of the REL2 immune pathway in the malaria mosquitoes should advance our knowledge of conserved mechanisms of innate immunity and may identify new targets for vector-mediated malaria control.

## Author contributions

All authors listed, have made substantial, direct and intellectual contribution to the work, and approved it for publication.

### Conflict of interest statement

The authors declare that the research was conducted in the absence of any commercial or financial relationships that could be construed as a potential conflict of interest.
